# African Elephant Alarm Calls Distinguish between Threats from Humans and Bees

**DOI:** 10.1371/journal.pone.0089403

**Published:** 2014-02-26

**Authors:** Joseph Soltis, Lucy E. King, Iain Douglas-Hamilton, Fritz Vollrath, Anne Savage

**Affiliations:** 1 Education and Science Department, Disney’s Animal Kingdom, Lake Buena Vista, Florida, United States of America; 2 Save the Elephants, Nairobi, Kenya; 3 Department of Zoology, University of Oxford, Oxford, United Kingdom; 4 Conservation Department, Disney’s Animal Kingdom, Lake Buena Vista, Florida, United States of America; Rutgers University, United States of America

## Abstract

The Samburu pastoralists of Northern Kenya co-exist with African elephants, *Loxodonta africana*, and compete over resources such as watering holes. Audio playback experiments demonstrate that African elephants produce alarm calls in response to the voices of Samburu tribesmen. When exposed to adult male Samburu voices, listening elephants exhibited vigilance behavior, flight behavior, and produced vocalizations (rumbles, roars and trumpets). Rumble vocalizations were most common and were characterized by increased and more variable fundamental frequencies, and an upward shift in the first [F1] and second [F2] formant locations, compared to control rumbles. When exposed to a sequence of these recorded rumbles, roars and trumpets, listening elephants also exhibited vigilance and flight behavior. The same behavior was observed, in lesser degrees, both when the roars and trumpets were removed, and when the second formants were artificially lowered to levels typical of control rumbles. The “Samburu alarm rumble” is acoustically distinct from the previously described “bee alarm rumble.” The bee alarm rumbles exhibited increased F2, while Samburu alarm rumbles exhibited increased F1 and F2, compared to controls. Moreover, the behavioral reactions to the two threats were different. Elephants exhibited vigilance and flight behavior in response to Samburu and bee stimuli and to both alarm calls, but headshaking behavior only occurred in response to bee sounds and bee alarm calls. In general, increasingly threatening stimuli elicited alarm calls with increases in *F*_0_ and in formant locations, and increasing numbers of these acoustic cues in vocal stimuli elicited increased vigilance and flight behavior in listening elephants. These results show that African elephant alarm calls differentiate between two types of threat and reflect the level of urgency of threats.

## Introduction

Mammalian vocalizations often refer to external objects or events in the environment, a phenomenon referred to as “referential” communication [1]. In many cases, mammalian vocal responses vary acoustically in the presence of different predators or predator classes, and listeners react to these calls as if they were in the presence of such predators. For example, vervet monkeys, *Cercopithecus aethiops*, usually respond to leopard alarm calls by running into trees, to eagle alarm calls by looking up, and to snake alarm calls by looking down [2]. Similarly, meerkats, *Suricata suricatta*, respond to aerial predator alarm calls by freezing, scanning and running for cover, and to terrestrial predator alarm calls by moving towards the sound source while scanning the area [3].

This research suggests that the acoustic features of calls can be related to specific external events, and that listeners can in turn act upon these acoustic features in adaptive ways. The variation in acoustic cues can be seen in examples taken from three species of *Cercopithecus*, in which vervet monkeys, *C. aethiops*, separate alarm calls by the location of dominant frequencies [2], Campbell’s monkeys, *C. campbelli*, separate them by call duration, and by the location and dynamic changes in dominant frequencies [4], while Diana monkeys, *C. diana*, separate them by call duration, fundamental frequency, and formant frequency characteristics [5]–[7].

Mammalian alarm calls are not always predator-specific. For example, yellow-bellied marmot, *Marmota flaviventris*, alarm calls are similar across a range of predators, but increase in rate with level of perceived risk [8]. Similarly, the behavioral responses of Belding’s ground squirrels, *Spermophilus beldingi*, vary according to predator type, but their vocal responses mainly reflect the severity of the threat [9]. It is likely that in many cases, alarm calls can refer to the predator type and the level of threat simultaneously. For example, meerkats, *Suricata suricatta*, produce distinctive alarm calls in response to aerial and terrestrial predators, but the acoustic structure of the calls also varies according to the degree of urgency within predator classes [3]. Predator class was distinguished by dominant frequency location, and urgency was reflected by call rate and degree of harmonicity [10].

African elephants, *Loxodonta africana*, have relatively few predators that threaten their survival in the wild, but known threats include humans and lions. Humans pose a variety of threats to elephants, including systematic poaching for ivory (e.g., [11]–[13]), habitat encroachment [14], and direct conflict over resources [15]. Importantly, elephants appear to recognize the level of threat that different human groups or different geographic areas pose. Fearful, defensive, and aggressive responses were observed in elephants when subjected to olfactory and visual cues of Masaai pastoralists, who are known to kill elephants, but the animals reacted less to olfactory and visual cues of Kamba agriculturalists, who pose less of a threat [16], [17]. Also, elephants spend less time and move more quickly through dangerous, non-protected areas, compared to less dangerous, protected areas [18], and elephants often avoid areas of persistent human habitation [17]. Elephants are also susceptible to predation by lions, calves being the most vulnerable [19; also see sources in 20], and playbacks of lion roars to female families resulted in defensive bunching behavior and matriarchal defense of the group [20].

In response to threats from predators, elephants are known to produce a variety of vocalizations, including rumbles, roars and trumpets [21], but until recently the alarm call system of the African elephant has received little systematic attention. Playback experiments by King et al. [22], [23] have shown that elephants run from the sounds of disturbed bees and also produce alarm calls that warn other elephants of the threat. In order to investigate further the alarm call system of the African elephant, we conducted a new series of experiments with the same methodology, but using a different threatening stimulus, the voices of Samburu tribesmen. The Samburu are pastoralists of Northern Kenya [24]. Their cultural attitudes and beliefs regarding elephants have traditionally limited the exploitation of elephants in terms of deliberate poaching for ivory or meat, but they do experience direct conflict with elephants, for example, at watering holes and during chance encounters in the bush, which sometime can be deadly [25], [26].

In the first experiment, we played the voices of male Samburu tribesmen to resting African elephants in the Samburu and Buffalo Springs National Reserves, Kenya, and recorded their behavioral and vocal responses. In a second experiment, we played the recorded vocal responses to resting elephants in order to examine their potential function as alarm calls. We played one natural and two experimentally modified sequences of calls, in order to explore the acoustic cues responsible for behavioral responses in listeners. We also present previously published and newly analyzed data from our previous experiments [23]. These data allowed us a) to show that African elephants produce alarm calls that differentiate between two types of threat (human versus bee), and b) to map the linkage between specific threats and the acoustic features of alarm calls, and between the specific acoustic features of alarm calls and the behavioral responses of listeners.

## Results

### Behavioral Response to Samburu Voice and Bee Sound Playbacks

We conducted 14 adult male Samburu voice playback trials on elephant families, consisting of a 2-min pre-stimulus phase, a 4-min Samburu voice stimulus phase, and a 2-min post-stimulus phase. For comparison, we provide results of 15 bee sound trials and 13 white noise control trials [23].

**Figure 1:**
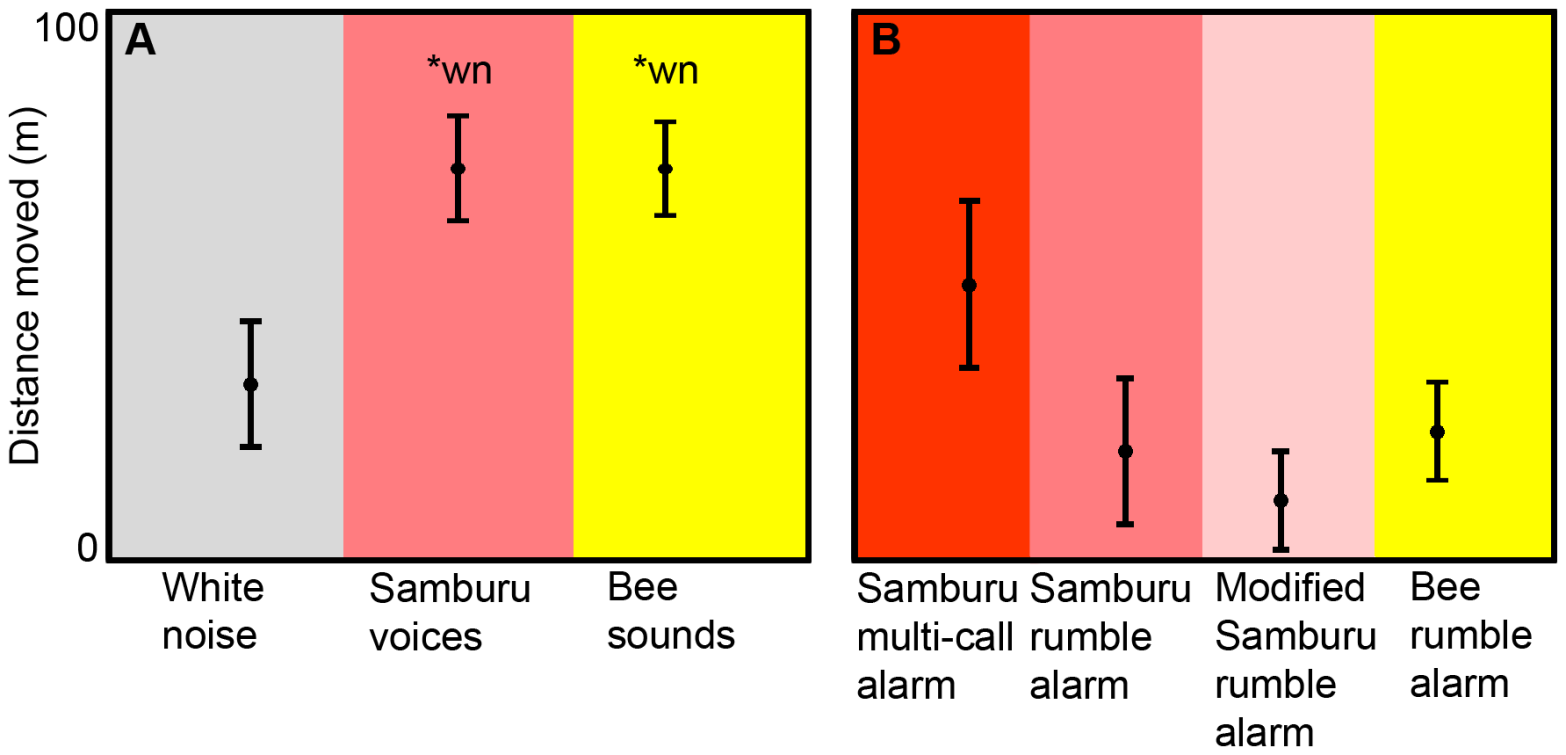
Distance moved from original sound playbacks and from vocalization playbacks. A) Distance moved (mean ± SEM) from playbacks of white noise controls (*n* = 13), Samburu voices (*n* = 14) and bee sounds (*n* = 15). B) Distance moved (mean ± SEM) from four vocalization playback stimuli (all *n* = 10). wn* = significantly different from white noise.

Samburu voices and bee sounds both elicited flight responses in elephant families (Fig. 1A; Table 1). Distance moved varied across the three playback stimuli (χ^2^ = 8.3, *df* = 2, *p* = 0.016), with greater distances observed in response to Samburu voices and bee sounds, compared to white noise (Samburu vs. white noise: *U* = 41, *n*_1_ = 14, *n*_2_ = 13, *p* = 0.014; bee vs. white noise: *U* = 45, *n*_1_ = 15, *n*_2_ = 13, *p* = 0.015). Distance moved in response to Samburu voices and bee sounds was similar (*U* = 102, *n*_1_ = 14, *n*_2_ = 13, *p* = 0.914).

**Table 1:** Behavioral responses to original sound playbacks.

Behavioralvariable	Playback	Response (mean ± SEM)		
Distance moved (m)	White noise	32.3±11.5		
	Samburuvoices	71.8±9.6		
	Bee sounds	71.7±8.5		
		**Pre-stimulus**	**Stimulus**	**Post-stimulus**
Vigilance (per min)	White noise	0.27±0.27	0.65±0.24	0.38±0.23
	Samburuvoices	0	2.02±0.36	0.43±0.29
	Bee sounds	0.13±0.06	2.25±0.45	0.60±0.31
Headshake (per min)	White noise	0	0.04±0.03	0
	Samburuvoices	0.04±0.04	0.04±0.02	0
	Bee sounds	0.07±0.05	0.27±0.06	0.03±0.03
Call rate (per min)	White noise	0.15±0.07	0.48±0.17	0.65±0.23
	Samburuvoices	0.46±0.18	1.27±0.33	0.82±0.19
	Bee sounds	0.47±0.16	1.27±0.29	1.53±0.44

**Figure 2:**
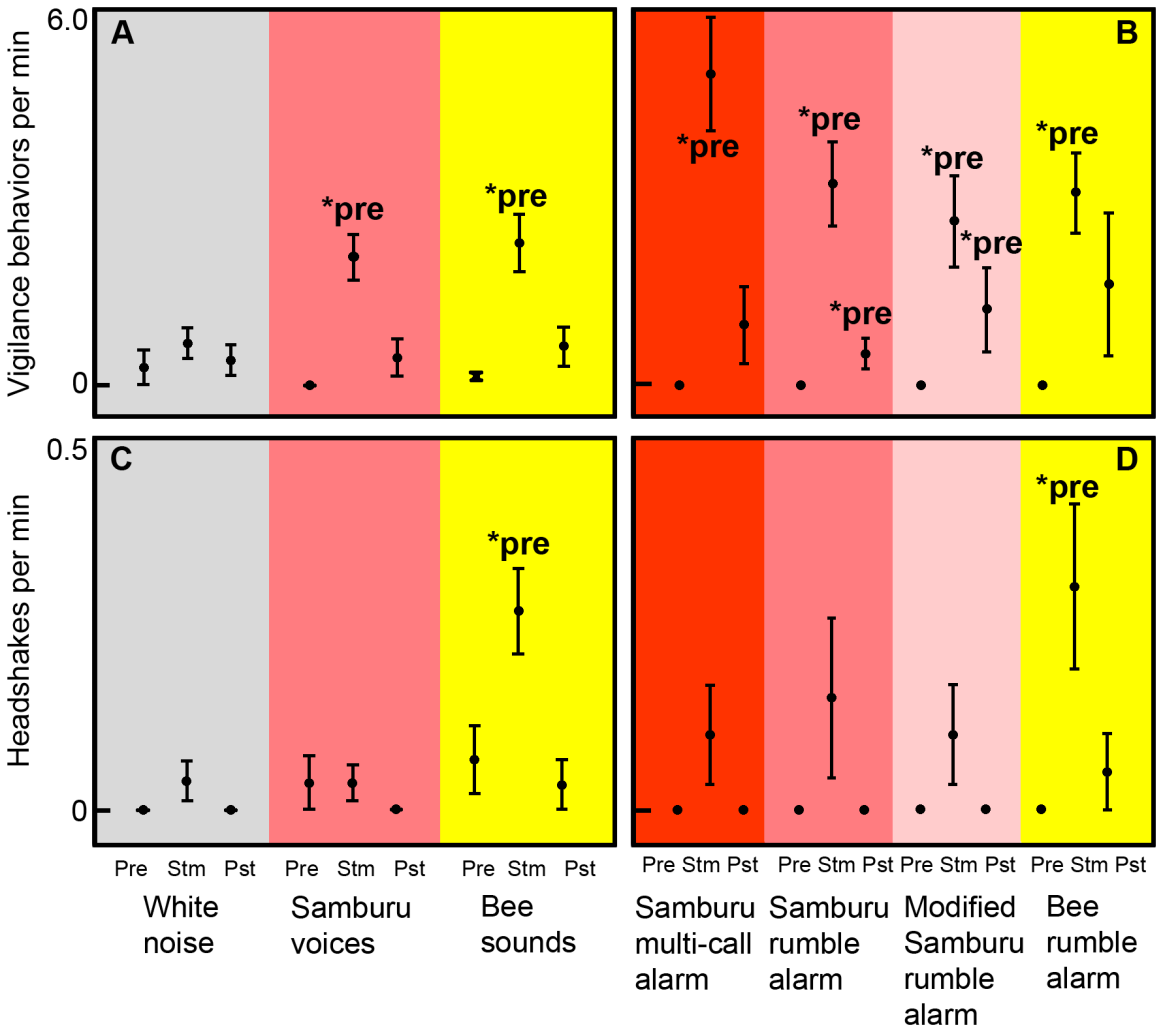
Behavioral response to original sound playbacks and to vocalization playbacks. A) Vigilance (mean ± SEM) across phases of playback trials for white noise (*n* = 13), Samburu voices (*n* = 14) and bee sounds (*n* = 15). B) Vigilance (mean ± SEM) across phases of playback trials for four vocalization playbacks (all *n* = 10). C) Headshaking (mean ± SEM) across phases of playback trials for white noise (*n* = 13), Samburu voices (*n* = 14) and bee sounds (*n* = 15). D) Headshaking (mean ± SEM) across phases of playback trials for all four vocalization playbacks (all *n* = 10). Pre = pre-stimulus phase; Stm = stimulus phase; Pst = post-stimulus phase. *pre = significantly different from pre-stimulus phase.

Samburu voices and bee sounds also both elicited vigilance behaviors (smelling, head-up, scanning) in elephant families (Fig.2A; Table 1). Vigilance varied across the three phases of Samburu voice (χ^2^ = 21.3, *n* = 14, *p*<0.000) and bee sound trials (χ^2^ = 19.0, *n* = 15, *p*<0.000), and in both cases vigilance was higher in the stimulus phase, compared to the pre-stimulus phase (Samburu voices: *Z* = −3.2, *n* = 14, *p* = 0.001; bee sounds: *Z* = −3.4, *n* = 15, *p* = 0.001). While vigilance varied across the three phases of white noise controls (χ^2^ = 7.7, *n* = 13, *p* = 0.021), no pair-wise comparisons were significant (all *p*>0.05).

In contrast to movement and vigilance behavior, headshaking behavior only varied across the three phases of bee sound trials (Fig. 2C; Table 1; χ^2^ = 10.9, *n* = 15, *p* = 0.004). Headshaking was higher in the stimulus phase compared to the pre-stimulus phase (*Z* = −2.3, *n* = 15, *p* = 0.001). On the other hand, headshaking was low and did not differ across phases of Samburu voice (χ^2^ = 2.0, *n* = 14, *p* = 0.368) or white noise trials (χ^2^ = 4.0, *n* = 13, *p* = 0.135).

### Vocal Response to Samburu Voice and Bee Sound Playbacks

**Figure 3:**
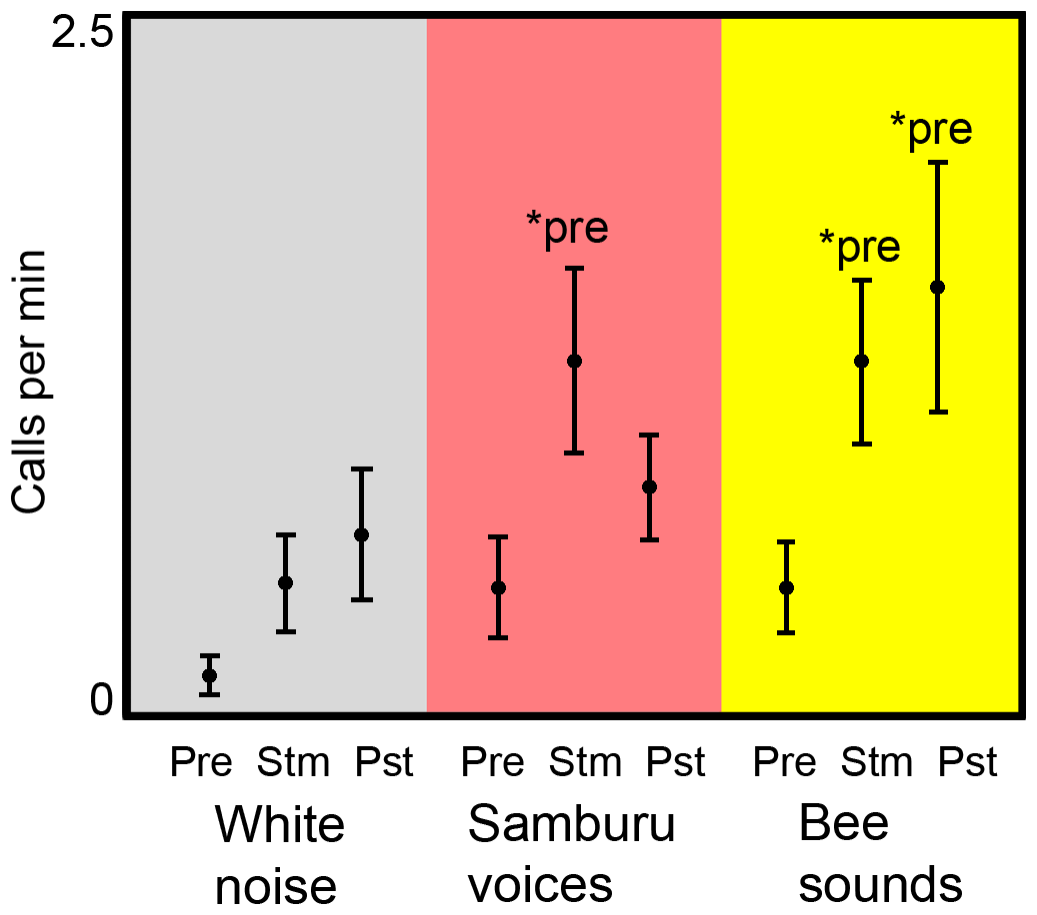
Call rate in response to original sound playbacks. Call rate (mean ± SEM) across phases of playback trials for white noise (*n* = 13), Samburu voices (*n* = 14) and bee sounds (*n* = 15). Pre = pre-stimulus phase; Stm = stimulus phase; Pst = post-stimulus phase. *pre = significantly different from pre-stimulus phase.

Samburu voices and bee sounds both elicited vocal responses from elephant families (Fig. 3; Table 1). Call rate varied across the three phases of playback trials for Samburu voices (χ^2^ = 8.4, *n* = 14, *p* = 0.015) and bee sounds (χ^2^ = 6.1, *n* = 15, *p* = 0.046), but remained low and did not differ across phases of white noise trials (χ^2^ = 4.3, *n* = 13, *p* = 0.118). In Samburu voice and bee sound trials, call rate was higher in the stimulus phase compared to the pre-stimulus phase (Samburu: *Z* = −2.7, *n* = 14, *p* = 0.007; bee: *Z* = −2.2, *n* = 15, *p* = 0.029). Additionally, call rate remained high in the post-stimulus phase of bee sound trials (*Z* = −2.3, *n* = 15, *p* = 0.024).

The rumble vocalization was the most common vocal response to Samburu voices (72/92 = 78%) and bee sounds (111/122 = 91%), in the stimulus and post-stimulus phases combined. Across contexts (responses during pre-stimulus control phases, and to Samburu voices and bee sounds), the acoustic structure of rumbles varied in terms of fundamental frequency (*F*_0_) mean (*χ^2^* = 17.5, *n*_1_ = 18, *n*_2,3_ = 20, *p*<0.001), *F*_0_ range (*χ^2^* = 14.0, *n*_1_ = 18, *n*_2,3_ = 20, *p* = 0.001), first formant (F1) location (*χ^2^* = 10.8, *n*_1_ = 18, *n*_2,3_ = 20, *p* = 0.004), and second formant (F2) location (*χ^2^* = 8.1, *n*_1_ = 18, *n*_2,3_ = 20, *p* = 0.017), but not for call duration (*χ^2^* = 2.2, *n*_1_ = 18, *n*_2,3_ = 20, *p* = 0.326).

**Figure 4:**
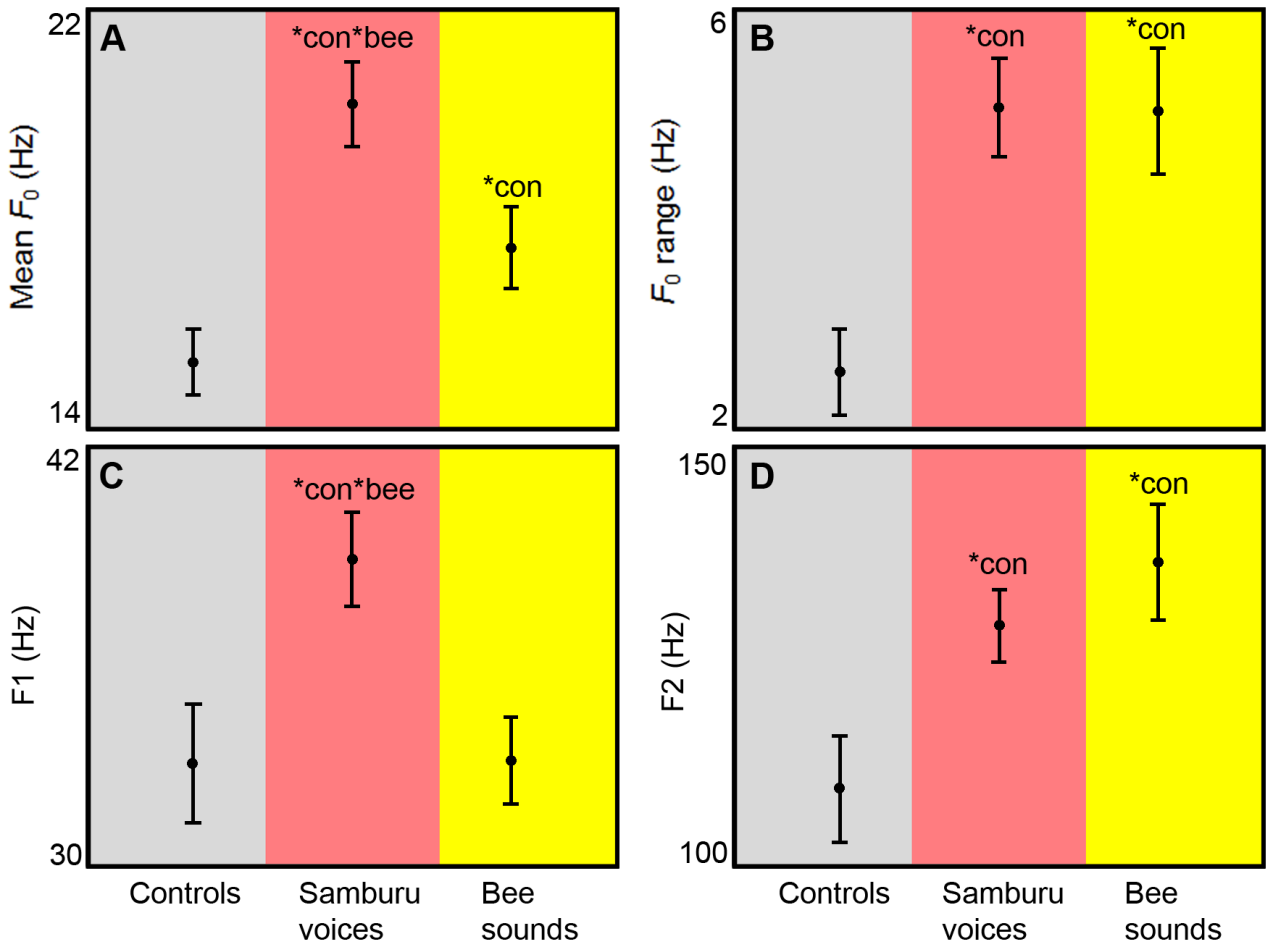
Acoustic structure of rumbles made in response to original sound playbacks. Acoustic features (mean ± SEM) of rumbles produced during pre-stimulus control phases (*n* = 18), and in response to Samburu voices (*n* = 20) and bee sounds (*n* = 20). A) Mean fundamental frequency (*F*_0_). B) *F*_0_ range. C) The first formant (F1) location. D) F2 location. *con = significantly different from controls. *bee = significantly different from bee sounds.

The acoustic structure of rumbles produced in response to Samburu voices was different than that produced in response to bee sounds (Fig. 4; Table 2). First, increases in mean *F*_0_ were observed in response to Samburu voices (*U* = 46, n_1_ = 18, n_2_ = 20, *p*<0.001) and to bee sounds (*U* = 102, *n*_1_ = 18, *n*_2_ = 20, *p* = 0.022), compared to pre-stimulus control rumbles, but the magnitude of increase was higher in response to Samburu voices compared to bee sounds (*U* = 111, *n*_1,2_ = 20, *p* = 0.015). Second, F1 location increased in response to Samburu voices compared to controls (*U* = 84.5, *n*_1_ = 18, *n*_2_ = 20, *p* = 0.004) and compared to bee sounds (*U* = 97.5, *n*_1,2_ = 20, *p* = 0.005), while F1 was similar in response to bee sounds and controls (*U* = 152.5, *n*_1_ = 18, *n*_2_ = 20, *p* = .426). Acoustic response was similar in terms of *F*_0_ range and F2 location, however, both of which increased in response to Samburu voices and bee sounds, relative to controls (*F*_0_ Samburu voices: *U* = 67, *n*_1_ = 18, *n*_2_ = 20, *p* = 0.001; *F*_0_ bee sounds: *U* = 72, *n*_1_ = 18, *n*_2_ = 20, *p* = 0.001; F2 Samburu voices: *U* = 100, *n*_1_ = 18, *n*_2_ = 20, *p* = 0.019; F2 bee sounds: *U* = 92, *n*_1_ = 18, *n*_2_ = 20, *p*<0.009).

**Table 2:** Acoustic structure of rumbles produced during pre-stimulus phases (controls), and in response to Samburu voices and bee sounds.

Acoustic variable	Rumble category	Response (mean ± SEM)
Mean *F*_0_ (Hz)	Controls	15.3±0.6
	Samburu voices	20.2±0.8
	Bee sounds	17.5±0.8
*F*_0_ range (Hz)	Controls	2.5±0.4
	Samburu voices	5.1±0.5
	Bee sounds	5.0±0.6
F1 location (Hz)	Controls	33.0±1.7
	Samburu voices	38.8±1.3
	Bee sounds	33.0±1.3
F2 location (Hz)	Controls	109.3±6.3
	Samburu voices	128.8±4.3
	Bee sounds	136.4±6.9

The acoustic changes in rumbles were not attributable to age or physical exertion. Across rumbles, acoustic variables were not significantly correlated with the age composition of the target family group (Spearman’s correlations, *n* = 58, all *p*>0.05) or distance moved away from Samburu and bee playback stimuli (Spearman’s correlations, *n* = 40, all *p*>0.05).

### Behavioral Response to Vocalization Playbacks

**Figure 5:**
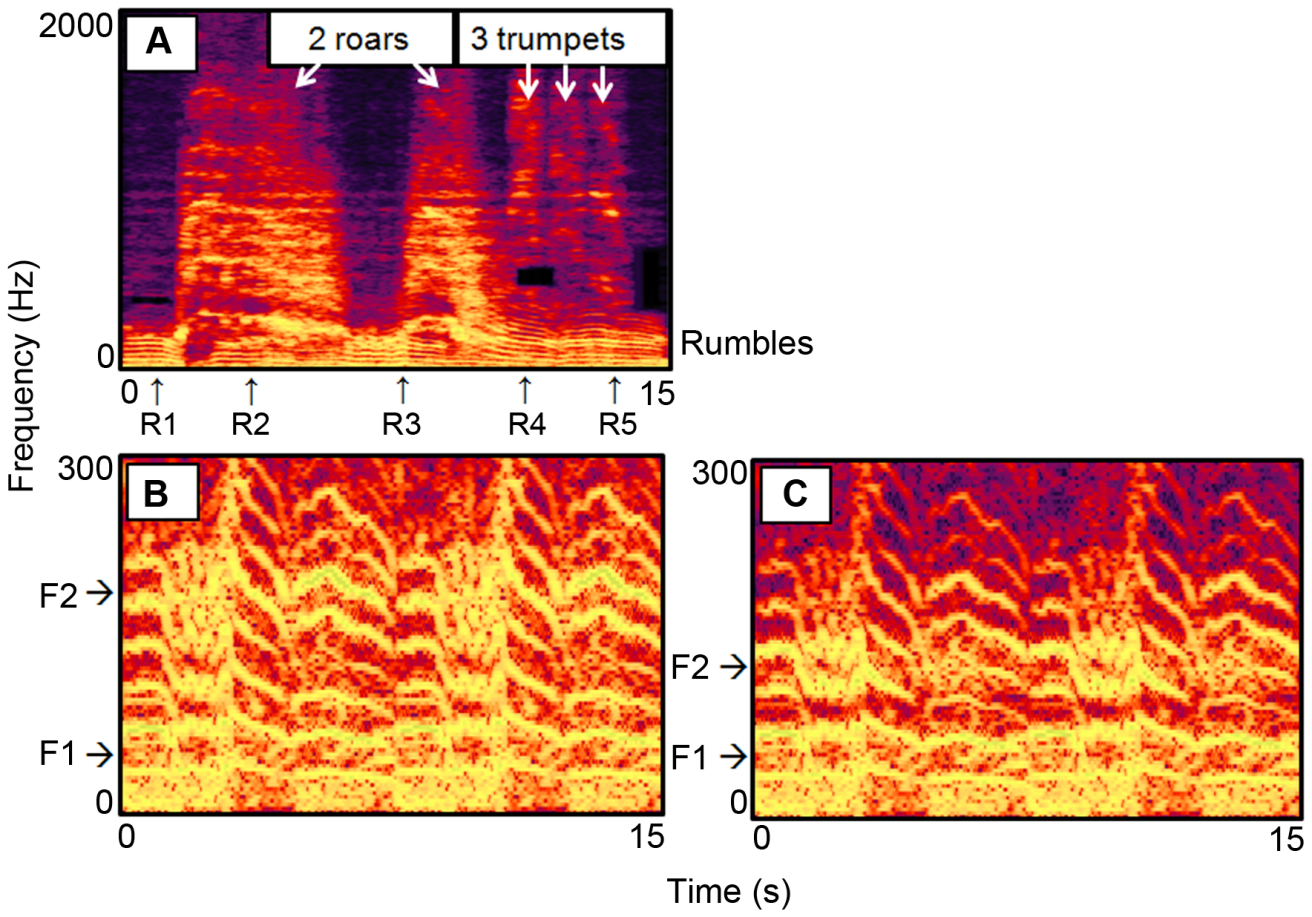
Spectrograms of elephant vocalization playback stimuli. A) Samburu multi-call alarm: unmodified vocal response to Samburu voice playback, with rumbles (black arrows) and roars and trumpets (white arrows). Nonlinear phenomena include chaos in roars, and bifurcation in one rumble (R3) and the second roar which transitions to a rumble (R4). B) Samburu rumble alarm: same as (A) but with roars and trumpets removed. Rumbles overlapping with roars (R2 and second half of R3) were simultaneously removed. The remaining rumbles were doubled. First and second formant (F1, F2) locations are indicated. C) Modified Samburu rumble alarm: same as (B) but with F2 lowered to resemble control rumbles. See Materials and Methods for details. Spectrograms were created in Adobe Audition (version 2.0, 44.1 kHz sample rate, frequency resolution = 8192 bands, Gaussian window).

We conducted a second playback experiment, consisting of a 2-min pre-stimulus phase, a 2-min vocalization stimulus phase, and a 2-min post-stimulus phase. Three different vocalization sequences, modified to exhibit decreasing levels of overall intensity, were played to elephants (Fig. 5): a) “Samburu multi-call alarm:” an extreme vocal reaction to the Samburu voice playbacks, which included rumbles, roars and trumpets, b) “Samburu rumble alarm:” a more typical response, which was the same call sequence as (a), but with roars and trumpets removed, and c) “modified Samburu rumble alarm:” the same call sequence as (b), but with the second formants artificially lowered to more closely resemble non-alarm rumbles. To determine if elephants produce specific alarm calls for different threats, we also present the behavioral reactions to rumble vocalizations that were produced in response to bee sounds (“bee rumble alarm;” [23]).

The three Samburu alarms and the bee rumble alarm elicited movement and vigilance behavior, but only the bee rumble alarm elicited headshaking. Elephant families moved away in response to all vocalization playbacks (Fig. 1B; Table 3), but the mean distance moved did not differ across the four vocalization playback stimuli (*χ^2^* = 6.0, *n*_1,2,3,4_ = 10, *p* = 0.112). Also, vigilance behavior increased across phases of playback trials for all vocalization stimuli (Fig. 2B; Table 3; Samburu multi-call alarm: *χ^2^* = 18.6, *n* = 10, *p*<0.001; Samburu rumble alarm: *χ^2^* = 18.6, *n* = 10, *p*<0.001; modified Samburu rumbles alarm: *χ^2^* = 11.6, *n* = 10, *p* = 0.003; bee rumble alarm: *χ^2^* = 14.0, *n* = 10, *p* = 0.001). Compared to pre-stimulus phases, vigilance increased in the stimulus phase for all vocalization stimuli (Samburu multi-call alarm: *Z* = −2.8, *n* = 10, *p* = 0.005; Samburu rumble alarm: *Z* = −2.8, *n* = 10, *p* = 0.005, modified Samburu rumble alarm: *Z* = −2.4, *n* = 10, *p* = 0.018; bee rumble alarm: *Z* = −2.7, *n* = 10, *p* = 0.007). Additionally, vigilance remained high in the post-stimulus phases for the Samburu rumble alarm (*Z* = −2.1, *n* = 10, *p* = 0.039) and the modified Samburu rumble alarm (*Z* = −2.2, *n* = 10, *p* = 0.026).

**Table 3:** Behavioral responses to vocalization playback stimuli.

Behavioral variable	Playback	Response (mean ± SEM)		
Distance moved (m)	Samburu multi-call	50.5±15.3		
	Samburu rumble	20.0±13.3		
	Samburu modified rumble	11.0±9.0		
	Bee rumble	23.6±8.9		
		**Pre-stimulus**	**Stimulus**	**Post-stimulus**
Vigilance (per min)	Samburu multi-call	0	4.95±0.90	0.95±0.61
	Samburu rumble	0	3.20±.68	0.50±0.25
	Samburu modified rumble	0	2.60±.72	1.2±0.67
	Bee rumble	0	3.05±.63	1.60±1.13
Headshake (per min)	Samburu multi-call	0	0.10±0.07	0
	Samburu rumble	0	0.15±0.11	0
	Samburu modified rumble	0	0.10±0.07	0
	Bee rumble	0	0.30±.11	0.05±.05

In contrast, headshaking behavior only increased during playbacks of bee rumble alarms (Fig. 2D; Table 3; *χ^2^* = 7.0, *n* = 10, *p* = 0.030), in which headshaking was higher during the stimulus phase compared to the pre-stimulus phase (*Z* = −2.1, *n* = 10, *p* = 0.034). Headshaking behavior was lower and did not differ across phases of any of the three Samburu alarm playbacks (Samburu multi-call alarm: *χ^2^* = 4.0, *n* = 10, *p* = 0.135; Samburu rumble alarm: *χ^2^* = 4.0, *n* = 10, *p* = 0.135; modified Samburu rumble: *χ^2^* = 4.0, *df* = 2, *p* = 0.135).

### Acoustic Properties of Elephant Vocalizations and Behavioral Response

Alarm call playbacks with acoustic features reflecting urgency elicited the strongest behavioral responses in listening elephants. In total, we have played 6 different vocalization stimuli to elephant families ([23]; present study), each with varying numbers of increases in fundamental frequency characteristics (*F*_0_, *F*_0_ range), formant frequency locations (F1, F2), and nonlinear phenomena (see Materials and Methods), compared to control rumbles (Table 4). Across the six playback stimuli, the number of these acoustic features that increased relative to controls was positively correlated with rate of vigilance behavior (ρ = 0.928, *n* = 6, *p*<0.008) and flight behavior (ρ = 0.812, *n* = 6, *p* = .050) in listening elephants, but was uncorrelated with headshaking behavior (ρ = 0.529. *n* = 6, *p* = .280; Table 4).

**Table 4:** Acoustic features of control rumbles and 6 vocalization playback stimuli, and behavioral responses to playbacks.

	Acoustic feature					Behavior	
	*F*_0_ (Hz)	*F*_0_ range (Hz)	F1 (Hz)	F2 (Hz)	NLP^a^	VIG^b^	DIS^c^
**Pre-stimulus control rumbles (**Mean +1 SEM)	15.9	3.0	34.7	115.6	NO		
**Vocal playback stimuli**							
Samburu multi-call mean	**23.1** d	**8.4**	**44.8**	**153.1**	**YES**	5.0	50.5
Samburu rumble mean	**21.2**	**5.2**	**51.9**	**145.3**	NO	3.2	20.0
Samburu modified rumble mean	**21.1**	**5.3**	**40.8**	100.9	NO	2.6	11.0
Bee rumble mean^e^	**16.2**	2.4	28.8	**132.1**	NO	3.1	23.6
Bee modified rumble mean^e^	**16.0**	2.5	29.4	104.2	NO	0.8	9.9
Control rumble mean^e^	14.9	**6.4**	31.9	114.5	NO	0.4	0.4

a Non-linear phenomena (NLP; See Materials and Methods) is a dichotomous variable (YES = present; NO = absent).

b VIG: Average rate of vigilance behavior (per min) in response to vocal playback stimulus.

c DIS: Average distance moved (m) in response to vocal playback stimulus.

d Values in **bold** are greater than +1 SEM of the pre-stimulus control rumble values.

e For further details on these playback stimuli, see [23].

## Discussion

### Alarm Call System of the African Elephant

These results show for the first time that African elephant vocalizations can function as referential signals. First, when exposed to Samburu voices or bee sounds, vigilance and flight behaviors were triggered, but only in response to bee sounds did headshaking behavior increase, compared to controls (Figs. 1&2). Second, the alarm rumbles for Samburu tribesmen and bees were acoustically distinctive. Most importantly, Samburu alarm rumbles exhibited increases in F1 and F2 location, while bee alarm rumbles only exhibited an increase in F2 (Fig. 4). Third, alarm calls for Samburu and bees elicited different patterns of behavior that paralleled the behavioral responses to the original sound stimuli. In each alarm call, vigilance and flight behaviors were triggered, but headshaking increased only in response to the alarm calls for bees, not to the alarm calls for Samburu tribesmen (Figs. 1&2).

While vigilance and flight behaviors may be adaptive for a wide variety of external threats, headshaking behavior may be a specific adaptive response to bees, namely, to knock bees away from the facial area. Headshaking can occur in more general contexts, such as when an elephant is agitated [27], but in these alarm call contexts headshaking appears to be a specific response to bees, as the behavior was observed only in response to bee sounds and bee alarm calls, not in response to any other original stimulus or vocalization playback (Fig. 2; [23]).

The results presented here also suggest that African elephant alarm calls reflect the urgency of threats. Generally, increases in call rate, *F*_0_ characteristics and in formant frequency locations were weakest in response to white noise controls, intermediate in response to bee sounds, and strongest in response to Samburu voices (Figs. 3&4; [23]), reflecting increasing levels of potential threat (unspecified threat from unfamiliar white noise, sting injury from bees, and sometimes deadly conflict with humans). Furthermore, the increasing level of urgency reflected in alarm calls also elicited increasingly strong behavioral responses in listeners (Table 4). Vocalization stimuli exhibiting only a simple increase in either absolute *F*_0_ or *F*_0_ variation produced only weak vigilance and flight responses in listeners, while vocalization stimuli that also exhibited increases in formant locations or nonlinear phenomena produced the strongest vigilance and flight responses in listeners. These results are consistent with the notion that specific acoustic characteristics of vocalizations can elicit affective responses in listeners [28]. In particular, high *F*_0_ and nonlinear phenomena in vocalizations are known to be arousing to listeners [29], [30], and may have contributed to the behavioral response to the vocal stimuli observed here.

### Acoustic Cues to Threat Type and Urgency Level

The acoustic features of elephant alarm calls represent separate types of threat (bees versus Samburu tribesmen) and reflect level of urgency. One interpretation of these findings is that filter-related features of calls (i.e., F1 and F2 locations) represent specific types of threat, while source-related features (e.g., *F*_0_ characteristics) reflect the level of urgency. A similar pattern exists in meerkats, in which dominant frequency locations distinguished threat type, while call rate and *F*_0_ characteristics reflected the urgency of the threat [10]. In fact, formant frequency and dominant frequency locations are common acoustic features that differentiate alarm calls in mammals ([2], [4], [7], [10], present study). In contrast, tempo-related (e.g., call rate) and source-related (e.g., *F*_0_) features often indicate levels of general arousal in mammals over a wide variety of contexts, ranging from social separations, bouts of aggression, to painful procedures [31]–[37]. However, it must be noted that this pattern is not universal, as tempo- and source-related features are also sometimes implicated in the differentiation of threat types [4]–[6], and filter-related features are also sometimes implicated in the vocal response to general arousal [33].

In African elephants, a similar pattern emerges. Filter-related features (F1, F2) differentiate the bee and human threat, while source-related features (e.g., *F*_0_, call duration, amplitude) are associated with a variety of arousing stimuli, including threats from other species, as well as during dominance interactions and other forms of social agitation ([23]; [38]–[42]; present study). However, shifting of F1 location was observed in adults during dominance interactions with social superiors [41], and formant shifts also occurred in infant elephants after nurse cessations [43]. It could be that infants have not yet developed active control of the vocal tract (see below), and that the F1 shift observed during adult dominance interactions constitutes an alarm call to elicit aid. More work will be needed to determine how source and filter features are related to threat type and level of urgency in African elephants.

### Mechanisms of Alarm Call Production

Variation in the acoustic structure of African elephant alarm calls can be influenced by mechanical effects along the entire vocal production pathway, from source effects via air pressure from the lungs and neural enervation, which influence vocal fold behavior, to filter effects of the supra-laryngeal vocal tract, which can enhance resonant frequencies (called formants) (see [44]–[46]). Herbst et al. [47] showed experimentally that the acoustic structure of rumble vocalizations can be produced from air pressure alone, which can increase *F*_0_ [45]. As the oscillation rate reaches the physical limit of the vocal folds, a sudden transition from regular to irregular oscillatory regimes may occur, resulting in nonlinear phenomena such as chaos and bifurcation (see Materials and Methods; [47], [48]). In fact, potentially distressful situations in elephants are known to produce increased *F*_0_ [38]–[41] and nonlinear phenomena [42], [49], [50]. The results presented here are also consistent with this pulmonary mechanism, as *F*_0_ increased with the level of threat posed (Fig. 4), and, in an extreme reaction to the human threat, presence of nonlinear phenomena was also evident (Fig. 5). Neural enervation of the vocal folds is also known to result in increased *F*_0_ [45], [51] and more variable *F*_0_ [45], [52]. Thus, the results presented here are consistent with pulmonary and neural mechanisms.

Effects of the vocal tract filter are also evident in elephant alarm calls. Stoeger et al. [53] have shown that elephants can produce rumbles nasally through the trunk and orally through the mouth, and that the formant frequency locations are lower in nasally produced rumbles (mean F1 = 40 Hz; Mean F2 = 169 Hz) compared to orally produced rumbles (mean F1 = 129 Hz; mean F2 = 415 Hz; also see [46], [54]. Based on these analyses, it is clear that the alarm rumbles reported here involve the trunk (Fig. 4), but the mechanisms involved in the subtle shifting of F1 and F2 locations are not known. In the Samburu alarm call, there was a simultaneous upward shift in F1 and F2 locations, which can be effected by simple shortening of the vocal tract [45]; [55]–[57]. In the bee alarm call, on the other hand, there was an upward shift in F2 location, but F1 location remained similar to controls (Fig. 4). In humans, vowel differentiation is largely affected by vocal tract manipulations, such as tongue placement, and independent shifting of formants is common [45], [58], [59]. Further work will be required to determine the mechanisms that produce independent formant-shifting in elephant alarm calls.

The formant-shifting observed in elephant alarm calls may be viewed as evidence of active vocal tract manipulation [7], as humans use active vocal tract manipulations to produce similar changes in formant locations, resulting in different vowel sounds and changes in word meaning [45], [58], [59]. As noted above, formant frequency and dominant frequency locations are common acoustic features that differentiate alarm calls in mammals ([2], [4], [7], [10], present study). Moreover, Fitch and Zuberbühler [60] review evidence showing that the behavior, anatomy and neural circuitry that underpin vocal behavior are broadly shared among humans and nonhuman primates. Taken together, these results suggest that active vocal control may be possible in nonhuman animals, in particular for nonhuman primates.

At present, it is unclear to what extent formant-shifting in elephant alarm calls is the result of voluntary vocal tract manipulations, the simple by-product of affective states, or some other mechanism (see [61]). However, the parallels between elephant vocal behavior and human linguistic abilities are suggestive. The independent modulation of formant locations distinguishes African elephant alarm calls, similar to the way in which such formant shifts distinguish vowels and word meaning in humans [45]. Also, elephants are known to exhibit vocal flexibility and vocal learning, by vocally imitating environmental sounds and the vocalizations of other species, including different elephant species and humans [62], [63]. Future work exploring these intriguing parallels between elephant and human communication will shed more light on the matter.

## Materials and Methods

### Ethical Statement

This research was reviewed from an animal welfare perspective by Disney’s Animal Care and Welfare Committee (approved 12 Dec 2007). Clearance for research was granted by the National Council of Science and Technology, Republic of Kenya (NCST/5/002/R/1189; 31 Dec 2006–31 Jan 2013).

### Samburu Voice Playbacks

We played the voices of Samburu tribesmen [24] to 14 elephant families (group size: 5–13) resting under trees in the Samburu and Buffalo Springs National Reserves, Kenya [64], [65]. Samburu voices were recorded from 7 adult male Samburu tribesmen who were on staff at the Save the Elephants’ research camp in the Samburu National Reserve. Two of the 7 tribesmen (29%) were part of the elephant monitoring program and their voices may have been familiar to local elephant families as they were often nearby elephants while in vehicles on patrol, but the other five tribesmen had no such habituating contact with elephants. A 1-min sequence that included talking (30 s) and singing and clapping (30 s) was used for playbacks. Talking and singing was conducted in their native Samburu language. Following previously published protocols [23], we performed playbacks from a camouflaged speaker (15–30 m from the nearest subject) in the dry season of February-March 2010. The speaker set-up was meant to simulate the sudden and unexpected presence of Samburu tribesmen nearby with no indication that they were in a vehicle (as elephants are habituated to vehicles). The research vehicle was always positioned such that the Samburu voices did not appear to come from the vehicle. Three audio-recording units were deployed in an array surrounding the target family to capture the elephants’ vocal response (44.1 kHz sample rate). Two units (Marantz PMD670 recorder, Earthworks QTC1 microphone, 4–40,000 Hz ±1 dB) were deployed from the research vehicle window in duffle bags (15–40 m from nearest subject). One unit (Marantz PMD671 recorder, Earthworks QTC50 microphone, 3–50,000 Hz ±3 dB) and a video recorder were deployed on the vehicle roof (20–30 m from nearest subject).

After set-up, a 2-min pre-stimulus phase began, followed by a 4-min stimulus phase and a final 2-min post-stimulus phase. The stimulus phase consisted of the 1-min Samburu voice sequence repeated 4 times. After each trial, the distance that the elephants traveled away from the sound source was estimated, using multiples of the known vehicle length as a guide (0–100 m; after 100 m, elephants were often out of view, so this was the longest possible distance scored [22]). The center of the elephant family was used as the starting and ending distance as elephants were bunched up under trees at the start of the playbacks and remained close when they fled from stimuli. Video of each trial was scored by a single observer (LEK observed all video data for this and the comparison study [22]) for group composition based on body size (age classes: 0–2 yrs, 3–14 yrs, >14 yrs) and the following behaviors: “Headshaking,” in which an elephant threw the head side-to-side by means of a slight twist to the neck that resulted in ears flapping through the air and slapping back onto the flanks of the shoulder; “Smelling,” in which an elephant raised the trunk into the air (sometimes called “periscoping”) or by extending the trunk directly out in front of its face; “Scanning,” in which an elephant, with ears held out, moved its head from a central position to the left or right and then back again to the center; “Head-up,” in which an elephant lifted its head upwards, with ears held out, and held that stance for more than two seconds. Smelling, scanning and head-up co-occurred with each other, so in these analyses they were summed and collectively referred to as “vigilance” behaviors.

The microphone array allowed for the identification of vocalizations produced by the target family, by comparing the relative amplitudes on the three microphones. Identification of individual callers was not possible. The number of calls recorded was 114 (rumbles = 91, roars = 6 and trumpets = 17). As in our previous playback experiments [23], field observations suggested that infants vocalized at random across playback trials, so we removed infant rumbles (0–2 yrs) from the analyses. We identified infant rumbles based on acoustic data from African elephants at Disney’s Animal Kingdom (0–3 yrs; n = 120 rumbles), in which infants aged 0–2 yrs produced rumbles with mean fundamental frequencies above 20 Hz and mean durations below 1.5 sec. Rumbles meeting both criteria (*n* = 7) were removed from these analyses. Less is known about the age-related changes of roars and trumpets so none of these calls were removed from the data set.

### Acoustic Measurement

Acoustic measurement followed previously published protocols [23]. Rumbles were cut from call start to call end in Adobe Audition (version 2.0) and acoustic measurement was conducted in PRAAT (version 5.2.22) using automated routines. Elephant rumbles were low-pass filtered (200 Hz cut-off, 10 Hz smoothing, Hanning window) and down-sampled to a 400 Hz sample rate to analyze low frequencies. For each call, the pitch floor and pitch ceilings were adjusted to surround the observed fundamental frequency. From the fundamental frequency (*F*_0_) contour, the mean *F*_0_ and the *F*_0_ range (maximum *F*_0_ minus minimum *F*_0_) were calculated. Calls were high-pass filtered (10 Hz cut-off, 1 Hz smoothing, Hanning window) to remove background noise below the signal. A Fast Fourier frequency spectrum of the middle 0.5 sec of the call was generated (bandwidth = 200 Hz) and the first two formant frequency locations were extracted by LPC smoothing without pre-emphasis. Duration was defined as the length of the sound file. Amplitude measures were not taken due to variable and unknown distances between microphones and individual callers.

Signal-to-noise ratio was sufficient to make full measurement on 46 of 91 rumbles (51%). After removing infant rumbles (*n* = 7; see above), there remained 39 rumbles (5 pre-stimulus control rumbles, and 34 stimulus and post-stimulus rumbles). We added the five control rumbles to the 13 pre-stimulus control rumbles from our previous experiments [23] for a total of 18 pre-stimulus control rumbles. As in our previous experiments, we randomly selected 20 rumbles from the 39 stimulus and post-stimulus rumbles, in order to balance sample sizes. Thus, acoustic comparisons were conducted on a total of 18 pre-stimulus control rumbles, 20 rumbles made in response to bee sounds [from 23], and 20 rumbles made in response to Samburu voices. The bee response rumbles were obtained from 9 different families, and the control and Samburu response rumbles were each derived from 11 different families.

### Vocalization Playbacks

We conducted a second series of playback experiments to determine if elephant vocalizations produced in response to Samburu voices elicited behavioral reactions in listening elephants. In order to examine a broad range of vocal response, we chose a vocal response to Samburu voices that was very intense in terms of call type and acoustic features related to arousal or other alarm calls in elephants [23], [40], [42], [66], and experimentally manipulated the signal to decrease its intensity in two successive steps (Fig. 5). The first stimulus (the “Samburu multi-call alarm”) included high-frequency calls (roars and trumpets), and evidence of nonlinear phenomena [48], all of which are indicative of extreme arousal in elephants [42], [49], [66]. Nonlinear phenomena included presence of non-harmonic, chaotic elements (roars and trumpets) and sudden transitions between chaos and harmonic structure (bifurcation). This stimulus represented an extreme reaction to Samburu voices. The second stimulus (the “Samburu rumble alarm”) was the same as the multi-call alarm, but with the roars and trumpets removed. This stimulus represented a more typical vocal response to Samburu voices across the 14 trials. First, most vocal responses to Samburu voices did not include roars and trumpets (only 3 of 14 trials, 21%, included roars and trumpets). Second, vocal responses to Samburu voices exhibited source (*F*_0_, *F*_0_ variation) and filter (F1, F2) features that were higher than controls, and the “Samburu rumble alarm” showed the same increases relative to controls (See Table 4 and Figure 4). The third stimulus (“modified Samburu rumble alarm”) was the same as the Samburu rumble alarm, but with the second formant locations artificially lowered to better resemble non-alarm-call rumbles. This stimulus represents a relatively weak vocal response, as it is missing one feature typical of rumbles produced in response to Samburu voices and to bee sounds [23].

The Samburu multi-call alarm was extracted from a recording from a single Samburu voice playback trial, and consisted of 5 rumbles, 3 trumpets and 2 roars (duration = 15 sec; Fig. 5a). The following manipulations were conducted in Adobe Audition (version 2.0). The original multi-call sequence was low-pass filtered to remove sounds with frequencies above the signal (Butterworth filter, 5000 Hz cut-off, order = 6). To produce the alarm rumble sequence, the roars and trumpets were removed from the original stimulus. Roars were broadband sounds spanning many frequencies, so all frequencies were selected and extracted from the signal where roars occurred (which also removed 1 overlapping rumble, and part of one other rumble; Fig. 5A). Trumpets were high-frequency calls and were removed with a low-pass Butterworth filter (600 Hz cut-off, order = 57). The sequence of four remaining rumbles was doubled (for 8 rumbles total) to match the duration of the multi-call sequence (15 sec; Fig. 5B). The modified rumble alarm was produced by artificially lowering the second formants of the rumbles, following a general procedure used previously [23]. Across the entire signal, the 125–250 Hz band was reduced by 12 dB, the 87–125 Hz band was increased by 6 dB, and the 70–80 Hz band was reduced by 12 dB. These amplitude manipulations reduced the second formant location (measured across all calls) from 154.6 Hz to 103.1 Hz (Fig. 5C).

All three vocal stimuli were matched for amplitude for playback trials (Adobe Audition, version 2.0). All stimuli were played through an FBT MAXX 4A speaker (frequency response: 50–20,000 Hz). Re-recording of rumbles at 1 m showed amplitude loss below 50 Hz, but frequency components were produced down to 20 Hz. Mean amplitudes measured 1 m from the speaker were 99.0, 100.8 and 100.1 dB for the multi-call alarm, the rumble alarm and the modified rumble alarm, respectively (NADY DSM-1 Digital SPL meter, C-weighting, slow response). Speaker distance was also matched across vocal stimuli in the field playback trials. Speaker distance was always between 40 and 50 m, and the mean distance between the speaker and the nearest subject of the target family was 45.0, 46.0, and 45.5 m for the Samburu multi-call, the Samburu rumble, and the modified Samburu rumble alarm, respectively.

Vocalization playback experiments were conducted in the Samburu and Buffalo Springs National Reserves in the dry season of February-March, 2011. Vocal stimuli were played back in random order until each stimulus was played 10 times to family groups (group size ranges: Samburu multi-call alarm = 5–10; Samburu rumble alarm = 5–12; Samburu modified rumble alarm: 6–13), using methods described previously [23]. After set-up of the speaker, a 2-min pre-stimulus control phase began, followed by a 2-min stimulus phase in which the 15 sec vocal sequence was played three times through the speaker (at the beginning, middle and end of the 2 min phase), and a final 2-min post-stimulus phase. After each trial, the distance that the elephants traveled away from the sound source was recorded (0–100 m; see above). A minimum gap of 5 days was allocated before the same family was tested with an alternate sound. We attempted to play all three vocal stimuli to the same family groups, but were unable to do so in all instances because families move into and out of the reserves and cannot be regularly encountered. Video of each trial was used to score behaviors and age-composition of the family group (see above).

When examining the effects of a class of vocal stimuli on listeners using one vocal stimulus from the class, the observed response could be due to any number of acoustic characteristics of the stimulus, not the specific feature or features hypothesized to characterize the class [67]. One means of overcoming this problem [67], and the one we adopted here (also see [23]), is to produce multiple stimuli by manipulating experimentally the acoustic features of interest so that only those features vary between the stimuli. In our first manipulation, we removed those parts of the call sequence that were relatively high in frequency and contained nonlinear phenomena, leaving only low-frequency rumbles that were produced by the same family group. In the second manipulation, we chose a feature (high second formant location) that was a typical vocal response to Samburu voices and bee sounds [23], and experimentally lowered the formant location to that typically observed in non-alarm call rumbles in African elephants [23], [46]. By exposing listeners to these stimuli, we were able to isolate the effects of these particular acoustic features, by comparing responses to contrasting stimulus-pairs that were identical except for the specific acoustic feature that was experimentally manipulated.

Employing such experimental manipulations, we have now played 6 acoustically distinct stimuli to listening elephant families ([23]; present study), each with variable numbers of increases in *F*_0_, *F*_0_ variability, F1 location, F2 location, and presence of nonlinear phenomena, relative to vocal responses in pre-stimulus control phases. As a result of these manipulations, we were able to relate specific acoustic features of vocalizations to specific behavioral responses in listeners. To create a threshold above which an acoustic feature was considered increased relative to control rumbles, the acoustic features in each playback stimulus were compared to the same features in pre-stimulus control rumbles. If the value of the acoustic feature of the playback stimulus was greater than 1 SEM above the mean for control rumbles, then the acoustic feature was considered to be higher than controls. Nonlinear phenomena in the form of chaos (noisy, non-harmonic elements of calls) and bifurcation (sudden transitions between chaos and harmonic structure; [42]) were either present or absent and occurred in only one vocalization stimulus (Samburu multi-call alarm). Based on these analyses, the 6 playback stimuli contained one to five acoustic features above controls (Table 4), and these acoustic features were mapped onto the behavioral responses of listening elephants.

### Statistical Analyses

All analyses employed non-parametric tests with two-tailed alpha set at 0.05 (SPSS, vers. 18). Kruskal-Wallis tests (χ^2^ statistic) were used to compare movement behavior and acoustic response across three playback stimuli (white noise, bee sounds, and Sumburu voices), and if statistically significant, Mann-Whitney tests (*U* statistic) were used for pair-wise comparisons. Friedman tests (χ^2^ statistic) were used to compare behaviors across the three phases within playback trials (pre-stimulus, stimulus, and post-stimulus) and if significant, Wilcoxon tests (*Z* statistic) were used to test whether or not the stimulus and post-stimulus phases were different from the pre-stimulus phase. Spearman correlations (ρ coefficient) were used to test for relationships between acoustic features and behavioral variables.

The same audio stimulus was never played to the same family more than once, so all the data within stimulus classes are independent. We attempted to play all three vocalization stimuli to the same 10 families, but were unable to do so (see Materials and Methods). Nevertheless, 8 families were played at least 2 different playback stimuli, so the comparison groups could lack statistical independence if the behavioral response of these elephant families in one playback trial influenced their response in subsequent trials. For example, elephants may become habituated to or over-stimulated by repeated audio playbacks. However, we could find no evidence for such order effects. The difference between the first and last playback trial was not significant for distance moved (*Z* = −1.1, *n* = 8, *p* = 0.269), rate of vigilance behavior (*Z* = −1.7, *n* = 8, *p* = 0.090), or rate of headshaking (*Z* = −0.00, *n* = 8, *p* = 1.000). Similarly, there were no detectable order effects in our previous experiments [23]. It is also possible that order effects occurred across years, but we could not find evidence for such effects. For 21 elephant families played more than one stimulus across all playback trials, the difference between the first and last playback trial was not significant for distance moved (*Z* = −0.3, *n* = 21, *p* = 0.753), rate of vigilance behavior (*Z* = −1.3, *n* = 21, *p* = 0.197), or rate of headshaking (*Z* = −0.5, *n* = 21, *p* = 0.603). Families exposed to more than one stimulus showed a mixture of increased, decreased and no change in behavioral response when comparing the first and last playbacks. Since there was no systematic order effect (i.e., systematic hypo- or hyper-reactivity to playbacks), then the variable responses observed across playback trials were likely due to the variable acoustic properties of each playback stimulus (which were played in random order), and not to the fact that some families were exposed to more than one stimulus.

### Correction

In our re-analysis of the data in our previous paper [23], we discovered errors in Figure 2 and associated data. Specifically, corrections were as follows: Error bars in Figure 2 were standard deviations, not standard errors of the means. Also, the “bee pre” and “bee stim” values of Fig. 2A were corrected in the current paper. Importantly, these corrections did not result in any changes in the statistical significance of any tests from the previous publication, and therefore did not change any of the conclusions stated in that publication. Nevertheless, Figure 2 in the current paper and the associated data should be considered accurate when compared to Figure 2 in the previous report [23].
